# Implementation of Virtual Reality to Parent-Child Interaction Therapy for Enhancement of Positive Parenting Skills: Study Protocol for Single-Case Experimental Design With Multiple Baselines

**DOI:** 10.2196/34120

**Published:** 2022-05-20

**Authors:** Iza C A Scherpbier, Mariëlle E Abrahamse, Robert G Belleman, Arne Popma, Ramón J L Lindauer

**Affiliations:** 1 Amsterdam UMC Location University of Amsterdam, Child and Adolescent Psychiatry Amsterdam Netherlands; 2 Levvel, Academic Center for Child and Adolescent Pyschiatry Amsterdam Netherlands; 3 Faculty of Medicine University of Amsterdam Amsterdam Netherlands; 4 Computational Science Lab University of Amsterdam Amsterdam Netherlands; 5 Amsterdam UMC Location Vrije Universiteit Amsterdam, Child and Adolescent Psychiatry & Psychosocial Care Amsterdam Netherlands

**Keywords:** PCIT, virtual reality, single-case experimental design, positive parenting skills, disruptive behavioral problems, parenting, child, disruptive behavior, behavioral, mental health, mobile phone

## Abstract

**Background:**

Disruptive behavior is a common reason for young children to be referred to mental health care services worldwide. Research indicates that treatments for child disruptive behavior where parents are the primary agents of change are most impactful. Parent-Child Interaction Therapy (PCIT) is an effective parent management training program currently implemented in therapeutic settings within the Netherlands. Ongoing research into improving the effectiveness of PCIT is being done within these settings. To further promote the key elements of PCIT, this study focuses on creating the opportunity for parents to practice positive parenting skills more outside of the clinical setting by adding virtual reality (VR) as an additional homework element. PCIT has shown to make impactful long-term improvements in parental warmth, responsiveness, and the parent-child relationship. Through VR, parents practice the taught parenting skills out loud in the comfort of their own homes in VR scenarios. We expect that VR addition will innovatively increase the effectiveness of PCIT.

**Objective:**

This study aimed to evaluate the added value of VR to PCIT by using a multiple baseline single-case experimental design (SCED). We expect to find that PCIT-VR will ameliorate positive parenting skills. By implementing the VR element, we secondarily expected that meeting the skill criteria will be achieved sooner, treatment completion rates will increase, and the parent-child relationship will be better, whereas parental stress and child disruptive behavior will decrease.

**Methods:**

A total of 15 children (aged 2-7 years) with disruptive behavior and their parents will be followed throughout the PCIT-VR treatment. Using a multiple baseline SCED with 3 phases, 15 families will fill out questionnaires weekly, in addition to having pre- and posttreatment and follow-up measurements to monitor their positive parenting skills, child disruptive behavior, parenting stress, and VR progress. Moreover, quantitative information and qualitative interviews will be analyzed visually and statistically and summarized to provide a complete picture of experiences.

**Results:**

As of February 2021, 6 families have been enrolled in the study at the moment of submission. Data collection is projected to be completed in 2023. Quantitative and qualitative results are planned to be published in peer-reviewed journals, as well as being presented at national and international conferences.

**Conclusions:**

The SCED—with its phased design, randomization, and the opportunity to replicate and assess both individual and group treatment effects—and adaptability of the VR technology are the strengths of the study. The risks of increased type I errors, maturation effects, or technological failure will be mitigated with the right statistical support. This study aims to magnify the scope of the treatment through additional skill training, ultimately in support of routinely implementing VR within PCIT.

**International Registered Report Identifier (IRRID):**

DERR1-10.2196/34120

## Introduction

### Background

Disruptive behavior is one of the most frequent reasons for young children to be referred to child and adolescent mental health care services worldwide [1]. Statistics show that of the population in the United States, 7% to 9% of children have a diagnosed behavioral problem [2], and in Western culture, there is an increase in the prevalence of behavioral problems [3]. Behavioral problems at a young age have an effect on the child, as well as on his or her surroundings and society at large [4,5]. The long-term effects of untreated, externalizing, disruptive behavior includes school dropouts, peer rejection, developing antisocial personality disorders, higher public costs for health care and education, and both nonviolent and violent delinquency and criminality in adulthood [6-10]. However, protective factors include enhancing prosocial skills for children and fostering the mental well-being of parents [11]. Therefore, to prevent child disruptive behavior or intervene at an early stage, children need evidence-based programs that focus on ameliorating parental skills and diminishing child disruptive behavior. Research concludes that treatments for child disruptive behavior where parents are the primary agents of change have the most substantive evidence for effectiveness [12].

There are currently a few parent management training (PMT) programs that focus on improving parent-child interactions, which are being implemented worldwide [13]. Importantly, parenting is fundamentally linked to children developing life skills, and worldwide research into parenting programs has shown that developmental, emotional, behavioral, and health outcomes for both parents and children can improve as a result [14]. Within PMT programs, research has shown that programs based on the social learning theory are the most effective [12], of which Parent-Child Interaction Therapy (PCIT) is one [15]. PCIT is an evidence-based treatment that focuses on diminishing externalizing disruptive child behavior by strengthening parenting skills [16]. The therapy, where parents and children play together throughout a session, distinguishes itself from most other PMT programs through live coaching from the therapist, which focuses on increasing positive parenting skills [17].

PCIT has been well-researched pan culturally, with effect sizes varying between 49% and 59% [18]. Specifically in the Netherlands, PCIT has also been shown to be effective with higher effect sizes in a randomized controlled trial (RCT) comparing PCIT with a 10- to 15-session care as usual treatment (family creative therapy) available in the community mental health setting (mean 45.4, SD 3.6, for PCIT vs mean 34.0, SD 4.93 for family creative therapy; t_23_=6.25; *P*<.001) [19]. Although these effect sizes indicate that PCIT is an effective treatment, this study aims to focus on the gains that can still be achieved by allowing more families to benefit from PCIT by increasing the effect sizes, accessibility, and impact of the treatment by focusing on strengthening positive parenting skills.

Worldwide, dropout rates vary between 12% and 67% [20], and specifically in the Netherlands, studies indicate that there is high attrition and that parents have a hard time grasping the different parenting skills. In that study, 52% of parents who received PCIT dropped out [19]. Attrition rates worldwide seem to be attributed to parenting stress levels and treatment barriers [20]. For instance, single parent status, problem severity of the child, and low socioeconomic status are often attributed to attrition. However, there remains some discrepancy in the literature on what factors contribute to families dropping out, although many studies have attempted to identify predictive factors [21-23]. Nonetheless, one study found that treatment completers reported significantly fewer child behavioral problems and less parental stress at follow-up than treatment dropouts [24]. This indicates that treatment should focus on making parents feel more confident in their parenting, thus potentially diminishing parenting stress and, with that, dropout rates. In further support, a study found that treatment completers showed increasing positive parenting skills and decreasing negative parenting skills from before to after treatment [21]. In addition, positive parenting skills appear to be crucial for improving the parent-child relationship [7]. A meta-analysis on PCIT showed that when meeting the skill criteria is required for completion of treatment, child externalizing behavior is significantly lower than when this is not a requirement [18]. However, when PCIT is offered in a shorter form in community samples (eg, the *standard* 12-session PCIT or the 8-session PCIT-Home), comparable effect sizes can still be yielded for a reduction in disruptive behavior [18,25]. Nonetheless, if skill criteria are met when taking the traditional PCIT protocol into account, PCIT has been shown to make impactful long-term improvements in parental warmth, responsiveness, and effectiveness [17]. In addition, studies have shown that the performance and motivation of a skill can be improved by deliberately practicing that skill [26]. Moreover, parenting programs that incorporate the opportunity to practice new skills with their children are considered more effective interventions than those that do not [27,28]. This suggests that it could be beneficial for parents to practice the skills they learn in the therapy more intensely, thereby potentially reducing dropouts and magnifying the scope of the treatment. It has previously been shown that with live coaching feedback, positive parenting skills increase rapidly, whereas negative parenting behavior decreases rapidly, especially early in the intervention. These results indicate that early interaction change in positive parenting skill acquisition is related to more positive child welfare outcomes [29]. In addition, research indicates that a current obstacle in PCIT is that parents may have a hard time grasping parenting skills, which leads them to drop out because of the inability to fully understand and translate the parenting skills to their home setting [19]. Therefore, to bridge the gap between practicing the (Praise, Reflect, Imitate, Describe, and Enthusiasm [PRIDE]) skills in therapy and applying the skills in a home setting, this study will implement a virtual reality (VR) element. VR allows parents to practice skills regardless of whether their child is home or awake, meaning that the ability to practice skills is on demand whenever they have their smartphone device near them. Over the past years, VR has become increasingly popular within the mental health care sector, as it creates opportunities that would otherwise not be possible [30]. Thus far, research has shown that using technology in PMT programs maintains positive treatment outcomes as long as therapist contact is maintained [31]. Specifically, within PCIT, previously implemented technology augmentations, such as Pocket-PCIT, have been shown to lead to increased treatment completion rates. This indicates a promising development in using technology innovations in an attempt to tackle similar barriers to treatment engagement [28].

VR offers multiple advantages in the field of psychological research. First, a stimulus can be presented in 3 dimensions [30]. Second, a virtual environment can be manipulated and controlled to create scenarios for participants, whereas in a real-world environment, there are always variables present that cannot be controlled [32]. Third, VR technology is designed for immersion, meaning that the created virtual world becomes the real world for a moment [33]. Clearly, VR is an experience generator that opens up possibilities for creating experiences that would otherwise not be possible [33]. Thus far, in psychology and psychiatry, VR has been an effective tool in the treatment of anxiety disorders, posttraumatic stress disorder, schizophrenia, eating disorders, and substance abuse disorders [34]. It is most commonly used in exposure-based therapy and behavioral skills training [35]. Considering this study, we will focus on the use of VR for the behavioral skills training of parents. The goal is to teach certain skills to be applied in multiple environments [36]. When using VR for behavioral skills training in this study, the goal is to create an environment where the parent can additionally practice skills that they find difficult to use in the parent-child interaction. A study on the development of mindfulness skills for borderline personality disorder has shown that practicing mindfulness within a virtual environment creates the opportunity to generalize the skills to the natural context outside of therapy [37]. In addition, it has also been shown that practicing and training skills for dangerous situations, such as fire hazards in a virtual environment, are also effective [36]. These studies are precedents of how it is possible to apply VR to practice and train skills in a virtual environment. Thus, this study aims to implement VR as a low-threshold opportunity for parents to practice the skills they are taught in therapy, on their own time, within the context of their own home.

To date, no study has implemented VR to improve positive parenting skills in PCIT or in other PMT programs, although passive technological augmentations have been successful thus far [28,31]. However, considering that studies indicate that a current attrition obstacle in PCIT is that parents may have a hard time grasping the parenting skills, the addition of VR as a tool to practice skills, which can be generalized to a person’s natural context, seems to be a good addition to the therapy. Nonetheless, research suggests that VR should and could, in no way, replace the clinician but rather should be integrated into the therapy to augment the intervention [38]. Therefore, the implementation of VR in PCIT creates an accessible platform for parents to further grasp positive parenting skills, thus increasing the potential for PCIT to have an impactful and lasting effect. Consequently, rather than VR being an additional layer of homework—and thus an additional life stressor—it may, in fact, alleviate parents as their ability to practice skills is essentially always *on demand*. The research group involved in this project previously tested this VR concept for PCIT with a test 360° video showing the PCIT skills [39]. This was consequently evaluated by PCIT therapists and researchers. All therapists and researchers were enthusiastic about the prospect of adding this VR element to the therapy, creating an additional opportunity for parents to practice [39]. For example, if parents are divorced and do not always have their child with them, they can practice their parenting skills nonetheless through VR. Although a double-blind RCT might be feasible in PCIT, there are significant limitations to implementing this design in clinical practice. For example, it is difficult to include a sufficiently large study population to say something meaningful about the additional VR element in clinical practice. Instead, we have chosen to use an innovative trial design, the multiple baseline single-case experimental design (SCED). This design will enable all participants to benefit from the additional VR element, which will consequently improve the power of the study compared with a traditional RCT. The design is further explained in the *Study Design* section. Therefore, the purpose of implementing PCIT-VR is to provide all parents with an easy opportunity to solidify their positive parenting skills in the comfort of their own homes.

### Aims

The primary goal of this research project is to evaluate the implementation of VR in PCIT. We expect to find that PCIT-VR will ameliorate positive parenting skills, leading to faster achievement of the meeting of skill criteria. We believe that if positive parenting skills are trained more frequently by implementing the VR element, additional effects will also take place, such as achieving child-directed interaction (CDI) skill criteria sooner than when not using VR and increasing the treatment completion rate. We expect that by implementing a VR element, parents benefit from more intensive training, and VR will additionally innovatively magnify the scope of families (eg, split families with separated parents) who can benefit from PCIT. In addition, parental stress, child disruptive behavior, and analytics of VR will be secondarily measured.

As a whole, PCIT-VR is developed as an augmented version of PCIT, where the focus lies on ensuring that parents practice and learn positive parenting skills intensely. This is achieved by incorporating practicing with VR as an integral part of the therapy. The threshold for practicing skills at home using VR is low, as parents are simply able to use their smartphone devices. PCIT-VR creates the opportunity for parents to practice and become familiar with the skills they have been taught in the therapy sessions in the comfort of their own homes. The implementation of VR functions as additional *skill training*. Providing families with PCIT-VR at an early age and early stage can potentially prevent them from needing more intensive help at a later stage and can possibly minimize the impact of child disruptive behavior, not only in the long run but also during the therapy itself.

Secondary objectives are the effects that we expect because of the overall amelioration of positive parenting skills that cause both parental stress and child disruptive behavior to diminish. Therefore, we expect that the total number of PCIT sessions will diminish and that the overall completion of treatment will increase. In addition, family dynamics and competencies will be secondarily measured, meaning that we will be able to evaluate parental compliance in the use of VR and evaluate potential risk factors for dropout. We believe that parents choosing to use VR will show higher levels of compliance as they want to use an additional tool to learn the skills. Secondary objectives also include positive VR experiences and general therapy satisfaction. Furthermore, we expect that PCIT-VR will secondarily lead to a better quality of parent-child relationships. We expect that the effects of PCIT-VR are further maintained and engrained in the long term because of the additional skill training provided by the VR scenarios.

## Methods

### Participants

Children (aged 2-7 years) and their parents will be referred through community channels after seeking help or support. The research will be conducted in clinical practice in the Netherlands, where families first follow a standard intake procedure (which includes an intake interview, completion of a set of questionnaires, and being referred to the right area of expertise) to establish their need for help. After this, families can be included in the study and receive PCIT-VR if the following criteria are met:

- Disruptive behavior problems of the child are a reason for referral
- Children are aged between 2 and 7 years
- Parents speak Dutch or English

A total of 15 families will be included, preferably those who finish the treatment (including all treatment phases and measurement points).

As the design is a SCED, the calculations for the number of participants are based on the What Works Clearinghouse (WWC) [40] and information gained from previous PCIT research. The WWC standards dictate that there are 5 criteria that should be acknowledged in a SCED. One of these criteria states that the minimum number of participants necessary to demonstrate an intervention effect is 3 [41]. In addition, the American Psychological Association [42] states that to display the effectiveness of an intervention, a minimum of 9 replicated single-case studies should be performed. Furthermore, attrition rates according to the standard PCIT ranged between 34% and 77% [43]. Specifically, in the Netherlands, a previous study showed a 40% attrition rate [19]. Therefore, this study will strive to include 15 families, preferably those who finish the entire trajectory to (1) adhere to a minimum of 3 participants according to WWC standards, (2) be able to state that the intervention is effective in accordance with the American Psychological Association guidelines, and (3) compensate for the potential of a 40% attrition rate.

### Inclusion Criteria

Families that are referred for treatment to the clinical practice will be screened using a questionnaire for behavioral problems, the Eyberg Child Behavior Inventory (ECBI) [44] and a questionnaire about parental stress (*opvoedingsbelasting vragenlijst* [OBVL]; in Dutch) [45]. All families that are referred will be required to fill out both questionnaires on the web, where their scores are checked to see whether they meet the inclusion criteria. The clinical cutoff score for the ECBI is >131 according to US norms, meaning that all scores >131 will be included [46]. For OBVL, a T score of ≥60 is considered problematic or even clinical, thus counting as the cutoff score.

### Exclusion Criteria

A potential participant who meets any of the following criteria will be excluded from participation in this study:

1. A child with severe physical impairment, such as deafness
2. A child with a mental disability (IQ<60)
3. An unsafe home situation, where home displacement is indicated
4. Child or parent with problems requiring personal health care, such as suicidality, or parents with problems with aggression regulation or addiction
5. Parents known to have severe problems with motion sickness

It should be noted that IQ will not be tested during the screening; rather, a clinical judgment will be made by the therapist or professional during intake.

### Study Design

The study will use a nonconcurrent multiple baseline SCED across the 15 participants. This means that 15 individual cases will be analyzed following the same design structure. The design has been reported according to SPENT (Standard Protocol Items: Recommendations for Interventional Trials extension for N-of-1 trial protocols) guidelines [47]. SCEDs are often implemented to determine whether a causal relationship exists because of the introduction of an independent variable to the dependent variable [48]. In addition, it is frequently used when attempting to answer questions regarding the addition of a component to an intervention [49]. In terms of this study, this means that we will evaluate whether the addition of a VR element will increase positive parenting skills through multiple baseline SCEDs.

SCEDs can be used to both evaluate treatment effects for individuals and assess the efficacy of individualized treatments [48]. Although SCEDs are often compared with a case study design, there is a clear distinction: SCEDs require deliberate manipulation of an independent variable, and results from SCEDs are usually both visually and statistically analyzed [50,51]. Another clear distinction from other designs is that SCEDs differ from experimental designs that are based on comparing groups; the manipulated variable in a SCED refers to repeated measurements of an individual who is assigned to different treatments rather than the manipulated variable being evaluated by assigning multiple individuals to different groups [50].

**Figure 1:**
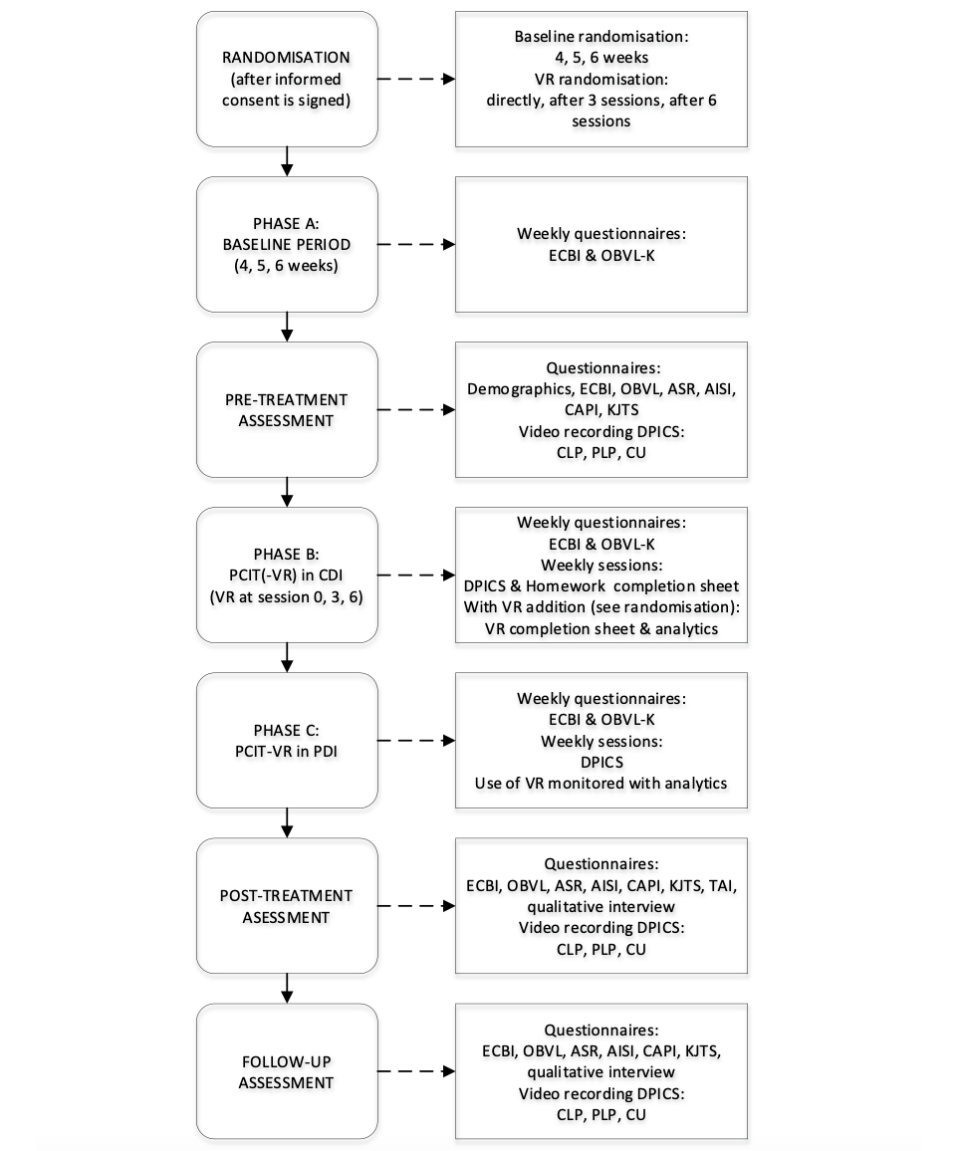
Graphical view with information per phase. AISI: Attachment Insecurity Screening Inventory; ASR: Adult Self-Report; CAPI: Child Abuse Potential Inventory; CDI: child-directed interaction; CLP: child-led play; CU: clean up; DPICS: Dyadic Parent-Child Interaction Coding System; ECBI: Eyberg Child Behavior Inventory; KJTS: Kind en Jeugd Trauma Screener; OBVL: opvoedingsbelasting vragenlijst; OBVL-K: opvoedingsbelasting vragenlijst-kort; PCIT: Parent-Child Interaction Therapy; PDI: parent-directed interaction; PLP: parent-led play; TAI: Therapy Attitude Inventory; VR: virtual reality.

**Figure 2:**
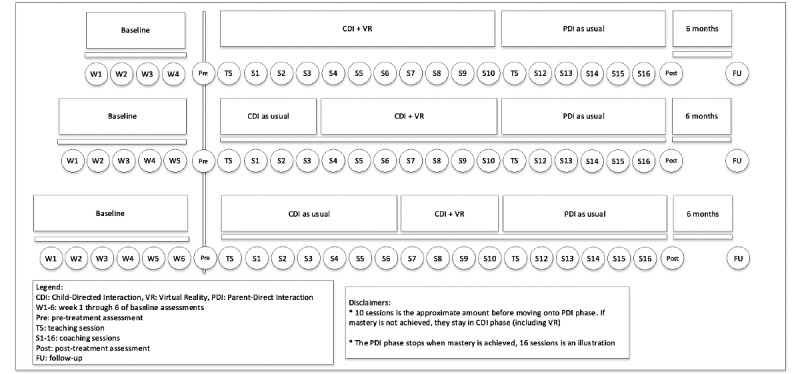
Three separate examples of the multiple baseline single-case experimental design in phases (using a fictitious number of allocated sessions).

Strong SCEDs can be characterized by three dimensions: (1) the design is divided into phases, (2) the design contains random assignment, and (3) the design should be replicated. Incorporating these dimensions improves the internal validity of the study and minimizes the possibility of history and maturation affecting the treatment effects [50]. These dimensions are incorporated into this study (Figures 1 and 2). First, the building blocks of a phase design can be divided into a baseline phase (A) and a treatment phase (B), with each phase containing multiple measurements. This can be made more complex by, for example, adding another treatment phase (C). Second, the phase design can be randomized by having different starting points for the treatment phase. In addition, a minimum of 3 measurement points is required per phase. In this study, randomization of the length of phases is used, which is increasingly considered important to make valid inferences about results. Third, the ways in which replication can occur is 2-fold: simultaneous replication entails conducting multiple phase designs at the same time, and sequential replication entails conducting multiple SCEDs to test the generalizability of the results. When both forms of replication are used, we speak of a hybrid design, which is called a nonconcurrent multiple baseline design, where partial temporal overlap is implied [50]. To adhere to these 3 dimensions and maximize internal validity, this study will use a nonconcurrent multiple baseline design.

The strength of SCEDs, when designed with phases, randomization, and replication, is that they allow for the comparison of an individual in different phases of treatment, thus demonstrating progression while also being able to compare the treatment effects of multiple individuals at different times [50]. Analyses are conducted both visually and statistically, thus providing an aggregated overview of individual and group treatment effects. SCEDs allow for a unique perspective where clinical practice lends itself to research rather than the other way round. An individual is personally followed, and data points are manipulated to provide results rather than their treatment conditions being manipulated. Subsequently, group comparisons can be performed on the basis of an individual’s own trajectory. Considering the fact that PCIT has already been widely researched, using the current design allows for an in-depth analysis of the newly implemented VR element in the treatment.

The multiple baseline SCED in this study is set up as shown in Figure 1. First, informed consent will be signed after families have received verbal and written information about the study. Second, participants will be dually randomized (by an external party) to determine their baseline period (4, 5, or 6 weeks of baseline measurements; phase A) and the addition of their VR element (VR at the start of CDI and after 3 or 6 sessions of CDI; phase B). After randomization, participants will start with phase A—the baseline period. Parents will electronically fill out the questionnaires weekly regarding child disruptive behaviors and parental stress using the ECBI and the short version of the OBVL. At the end of phase A, a pretreatment assessment will be performed. In this assessment, parents will be required to electronically fill out more questionnaires regarding both child and parental factors (Figure 1; see the *Assessment Instruments* section). In addition, the researcher will also make a video recording of the parent and child playing in 3 play situations (child-led play [CLP], parent-led play [PLP], and clean up [CU]), which will consequently be coded using the Dyadic Parent-Child Interaction Coding System (DPICS) [52]. Following this, in phase B, the intervention phase starts. Depending on their random allocation, families will start off with PCIT with or without the VR element. Once again, they will electronically fill out the ECBI and OBVL-*kort* (OBVL-K) weekly, and they will also register their homework completion and (if applicable) their VR completion sheets. During the PCIT sessions, PCIT therapists will register the parental progress weekly by coding 5 minutes of playtime with the DPICS. Once families reach CDI completion, they will move on to phase C, which is the parent-directed interaction (PDI) phase of PCIT. Measurements in phase C are identical to those in phase B. After treatment completion, a posttreatment assessment will be performed, which will be identical to the pretreatment assessment. Phase D is the follow-up assessment, which takes place 6 months after treatment completion. The assessment is also identical to the pre- and posttreatment assessments (Figure 2; 10 CDI sessions and 6 PDI sessions are used as a random example).

### Treatment

PCIT focuses on changing dysfunctional parent-child interactions in 2 phases and teaches authoritative parenting [17], which implies firm control, warmth to the parent-child relationship, and a balance between discipline and stimulating independence [53]. Therapists coach parents from behind a 1-way screen through an earpiece [17]. In the first phase of PCIT, which is the CDI phase, emphasis is placed on building up the quality of the parent-child relationship and increasing positive parenting skills. In addition, in this phase, children begin to build the ability to regulate their behavior [15]. This first phase lays the foundation for effective behavior change. In the CDI phase, parents follow their child’s lead in playing and are coached on using positive parenting skills, specifically, praise, reflective statements, behavioral descriptions, imitation, and enthusiasm [17]. The number of CDI sessions is dependent on the parents meeting the skill criteria (10 behavioral descriptions; 10 reflective statements; 10 labeled praises; and <3 questions, commands, or negative verbalizations during a 5-minute observation period).

However, the CDI phase alone is typically insufficient for children with disruptive behavior to return to displaying normal behavior [54]. After meeting the CDI skill criteria, the second phase of PCIT, which is the PDI, starts. The goal of the PDI phase is to further enhance the parents’ positive parenting skills to set consistent, predictable, and age-appropriate boundaries for their children [17]. In the PDI phase, parents are coached on using clear commands and how to apply consequences for compliance (praise) and noncompliance (timeout). In addition, they are taught and encouraged to keep using the positive parenting skills from the first phase. Once parents demonstrate achievement of skill criteria of both CDI and PDI skills, and their children’s behaviors according to the raw score on the ECBI Intensity Scale is <114, the treatment is completed, as standard protocol dictates [17].

In October 2020, 12 therapists were trained to become PCIT therapists at a clinical treatment center in the Netherlands. They were trained by PCIT International certified trainers, thus ensuring the quality of treatment. Therapists follow PCIT protocol and have biweekly consultations with their trainers to keep track of their progress. In addition, the clinical center already had multiple PCIT therapists trained approximately 10 years ago, who also still provide PCIT to families. Both newly trained and other PCIT therapists will help collect data for this study.

### VR Implementation

In the standard protocol of PCIT, parents are required to practice the skills taught throughout the sessions in *special time* at home with their children. This means that the families participating in PCIT have homework to do at home to improve weekly. This study suggests that parents also practice the skills they are taught in the sessions at home in the virtual environment that will be created for them. As such, the implementation of VR is an additional element to the standard PCIT protocol PCIT, as described previously.

There are multiple different systems within VR, one of which is a head-mounted display [30]. This study will be using 360° videos that can be played on a smartphone device placed on the head-mounted display. As a result, the person with the headset on can look around 360° from a singular point in the virtual environment, meaning that the environment does not change if the person wearing the headset decides to move. In technical terms, this is referred to as having 3 df. This option of technology was chosen as opposed to other forms of VR as it is inexpensive and easy to use through a simple (but protected) link, thus allowing people to access it from anywhere and at their own time, which increases accessibility and applicability.

The 360° video content for this study will be recorded using child actors for different scenarios. The various scenarios depict the positive parenting skills that are taught in the CDI phase (praise, behavioral description, reflective statement, and ignoring unwanted behavior), thus allowing parents to repeatedly practice these skills in the comfort of their own home. These verbalizations provide a variety of responses and depict scenarios with commonly used toys. As there are multiple scenarios with which the parent can practice, parents are free to choose the scenarios that most suit their goal of practice.

The recorded video will be edited into fragments containing key events that call for a response from the parent by answering a multiple-choice question asked at the end of each fragment. The fragments are assembled into a therapy scenario using a web-based scenario editor in which therapy is described by a nonlinear sequence of fragments, the order of which is determined by a parent’s responses. Coaching—in the way that the therapist would do in therapy sessions—is implemented by providing textual feedback to each given answer. The technology to support PCIT-VR is based on a web-based design that (1) provides a VR scenario editor, (2) functions as a server that delivers the content of a therapy scenario to the parent’s headset, and (3) records performance data for future analysis. The web-based design allows the therapy scenarios to be used on any device that runs on a web browser and allows use from any location with internet access.

### Assessment Instruments

The primary end point of positive parenting skills will be measured using the DPICS [52], and the added value of VR will be measured through VR assessments. Secondary end points, including the psychological functioning of the child, family dynamics and competences, and general therapy evaluation, will be measured through questionnaires and qualitative interviews. This is further explained in the following sections.

The DPICS [52] is a reliable and valid behavioral observational coding system designed to measure the quality of social interactions between parents and children. By coding open verbal and physical behaviors exhibited by both parents and children, the DPICS is able to assess the quality of the parent-child interaction as a construct. Behaviors that are coded are divided into the categories displayed in Textbox 1.

**Parent and child behaviors as coded in the Dyadic Parent-Child Interaction Coding System.**

- Parent behaviors
  Direct command: compliance, noncompliance, and no opportunity to comply
  Indirect command: compliance, noncompliance, and no opportunity to comply
  Question
  Behavioral description
  Reflective statement
  Labeled praise
  Unlabeled praise
  Neutral talk
  Negative talk
- Child behaviors
  Negative talk
  Prosocial talk
  Question
  Command
  Whine
  Yell

Coding during assessments will be done through 3 standard 5-minute play situations (CLP, PLP, and CU). Within the 5-minute observational videos, open verbal and physical behaviors will be scored on a score sheet. To progress within PCIT, parents must achieve 10-10-10 positive behaviors (10 behavioral descriptions, 10 reflective statements, and 10 labeled praises) and must display no negative leading behaviors (questions, negative talk, and commands in CDI) within the 5-minute observation time. At every assessment point during treatment, the therapist will score the parent during the 5-minute observation time with the DPICS. A video recording will be made of this, which will be used to assess the parents’ progression. In addition, an independent researcher will randomly select a few of the sessions’ video recordings and score the DPICS to assess the quality of the coding. Researchers will be trained beforehand to score the video recordings through DPICS and will be required to achieve an agreement rate of 80% on the scored behaviors. All observations will be transcribed verbatim to monitor interrater reliability and be subsequently double coded by an independent and reliable coder for 20% of the pretreatment, posttreatment, and follow-up treatment observations.

Further assessment instruments are divided into 4 subcategories and explained in the following sections.

### VR Evaluation

#### VR User Analytics

Each parent will have a personal sign-in that registers their answers when using VR. Their progress will be monitored any time they log in and use a therapy scenario.

#### VR Completion

The parents will be required to use a VR tracking sheet, where they will fill out the number of days that they had completed the VR training. This will then be divided by the total number of days in the week, yielding a percentage of VR completion per week. Parents will be required to fill this out weekly (alongside their *special time homework completion*, which is incorporated in the standard protocol of PCIT) as soon as they receive VR.

#### Qualitative Interview (Parents)

Parents will evaluate the learnability and usability of their VR experience. Questions reflect on how their experience with VR was, what they believe is the added value of VR to the therapy, and whether VR helped to strengthen and comprehend parenting skills at a faster pace. They will be asked qualitative questions about their VR experience during the posttreatment and follow-up assessments. In addition, questions surrounding whether the VR simulation is compatible with their manners of speaking to their children will also be asked.

### Psychological Functioning (Child)

#### Questionnaire Background Information

This questionnaire contains questions with regards to background information about the child and his or her family. Questions concerning sex, age, ethnicity, family composition, parental work situation, and education level are included. This questionnaire will be filled out at the pretreatment assessment. If necessary, their treatment file can be consulted for supplementary information.

#### Child Behavior Questionnaire: ECBI

The ECBI [44] is a questionnaire containing 36 items, which is filled out by parents and addresses child behavioral problems for children aged between 2 and 16 years. The ECBI is a part of the standard protocol of PCIT. The scale has good psychometric properties. Both the frequency of problem behavior (Intensity scale; 7-point scale from 1 [never] to 7 [always]) and the extent to which parents experience the behavior as problematic at that moment in time (Problem scale; yes or no) will be measured. Parents will complete the questionnaire at all assessment points.

#### Kind en Jeugd Trauma Screener

The Dutch translation of the Child and Adolescent Trauma Screen, which is the Kind en Jeugd Trauma Screener [55] screens for exposure to traumatic events and posttraumatic stress disorder symptoms. It is based on the Diagnostic and Statistical Manual of Mental Disorders, Fifth Edition, and contains 16 items in addition to 5 dichotomous questions about how symptoms limit their daily functioning. The psychometric values of the Dutch translation are currently being researched; however, according to the English, German, and Norwegian versions of the screening questionnaire, it is valid and reliable [55]. Parents will complete this questionnaire at the pretreatment and posttreatment assessments.

#### Child Abuse Potential Inventory

The Child Abuse Potential Inventory [56] is a questionnaire filled out by parents, which measures the chances that parents (physically) abuse their child (aged between 0 and 18 years). All 34 items include a statement with which parents could either *agree* or *disagree*. The psychometric properties of the Dutch translation have been shown to be reliable and valid [57]. This study will use the short version. Parents will fill out this questionnaire at the pretreatment and posttreatment assessments.

### Family Dynamics and Competences

#### Dutch Parenting Stress Questionnaire: OBVL

The OBVL [45] is a Dutch questionnaire with 34 questions that address five aspects of parenting stress: problems in the parent-child relationship, problems in parenting, depressed moods, role restriction, and health complaints. The total score on parenting burden can be calculated. The OBVL has high reliability, and norms have been measured for different age groups, indicating that its psychometric properties are good. Parents will complete this questionnaire during pre- and posttreatment and follow-up assessments.

#### Dutch Parenting Stress Questionnaire, Shortened Version: OBVL-K

The OBVL-K [45] is a shortened version of the OBVL. It comprises 10 questions that together provide a total score on the burden of parenting. This questionnaire has high reliability, and norms have been measured for different age groups, indicating that its psychometric properties are good. Parents will complete this questionnaire weekly to obtain a general overview of their parental burden throughout the process.

#### Adult Self-report

The Adult Self-Report [58] is a self-report measure of competencies and emotional and behavioral problems for adults and is based on the Diagnostic and Statistical Manual of Mental Disorders. The questionnaire is split into 2 sections, one concerning competencies and work and the second concerning emotional and behavioral problems. The English version has been shown to be reliable and has good validity. The psychometric properties of the Dutch version are yet to be evaluated. The parents will complete this self-report during pre- and posttreatment assessments.

#### Attachment Insecurity Screening Inventory

The Attachment Insecurity Screening Inventory (AISI) [59] is a questionnaire that measures attachment problems. The questionnaire contains 20 items that together form total attachment but are divided into three subcategories: avoidant, ambivalent/resistant, and disorganized attachment. The AISI 2-5 was found to be valid and reliable according to recent research [59]. The AISI 6-12 is practically identical to the AISI 2-5 but has not been tested for validity and reliability. Nonetheless, studies have shown that the AISI is a promising instrument for measuring attachment insecurity in young children [59]. The AISI will be filled out by parents during the pre- and posttreatment assessments.

### Therapy Evaluation

#### Therapy Attitude Inventory

The Therapy Attitude Inventory [60] is a 10-item scale that evaluates parental satisfaction with the treatment. This questionnaire will be filled out once by the parents at the end of the treatment. Although information regarding the reliability and validity of the Dutch translation of the TAI is missing, the original version has shown adequate psychometric properties [61].

#### Qualitative Interview (Parents)

A qualitative interview after treatment and follow-up assessments will allow parents to share their experiences with regard to the new form of PCIT-VR and the extent to which this therapy strengthened their competences and made them see their own abilities.

#### Qualitative Interview (Therapists)

Therapists will also be asked about their experiences and vision of the effectiveness of PCIT and their vision of the added value of VR to the therapy at the end of the data collection period. They will be asked what factors contribute to effectiveness, including factors such as their relationship with parents or specific characteristics. Through this interview, the different benefits and obstacles of PCIT-VR can be brought to light through the eye of the therapist.

### Statistical Analysis

Data analysis will take place in consultation with a statistics expert from the Clinical Research Unit department of the Amsterdam University Medical Center (UMC). Data analysis will include the following:

1. Primarily, the added value of VR to PCIT will be evaluated through analyses.
2. SCED analyses will be conducted with the Shiny app.
3. Repeated measures analyses will be conducted.
4. Graphical analyses will be conducted to render the change over time.

Qualitative information from the interviews will be summarized into a complete picture of the experiences of both parents and therapists. This will include their experiences with PCIT-VR, the VR component in particular, and how the therapy helped them gain insight into strengthening their competencies. All qualitative information will be analyzed using structured qualitative interview software, for which we will also seek consultation with an expert.

### Confidentiality of Data

The data for this study will be collected by researchers from the Department of Child and Adolescent Psychiatry at the Amsterdam UMC. The Clinical Research Unit of the Amsterdam UMC provides support for the data analysis of this study. All data will be stored in coded form by assigning personal numbers to each family and registering all information using that number. This ensures that only authorized people can trace which file belongs to which family. All study results will be published anonymously.

### Ethics Approval

This study was approved by the medical ethics committee of the Academic Medical Center of Amsterdam, Netherlands (2020_143). Informed consent for participation in the study will be obtained; among other permissions, parents will also provide permission for video recording of their child. The way informed consent will be obtained for the study was approved by the medical ethics committee of the Academic Medical Center of Amsterdam, the Netherlands (2020_143).

## Results

As of February 2021, 6 families have been enrolled in the study at the time of submission. Data collection is projected to be completed in 2023. Both quantitative and qualitative results are planned to be published in peer-reviewed journals, as well as being presented at national and international conferences. The study is registered at the medical ethics committee of the Academic Medical Center of Amsterdam, the Netherlands (2020_143/NL74210.018.20) and the Netherlands Trial Register NL9580.

## Discussion

### Principal Findings

This study is the first to implement a VR element that functions as additional *skill training* to magnify the scope of PCIT through practice. Similar to standard PCIT, the intervention is centered around parent-child play sessions, where parents receive live feedback and support from a therapist. The additional VR element provides parents with the opportunity to practice positive parenting skills in the comfort of their own homes without their child present while still receiving feedback. This allows them to further engrain the skills alongside the therapy sessions and special time. Therefore, we expect that the implementation of VR will lead to faster achievement of meeting the skill criteria. Furthermore, this study expects that parental stress, child disruptive behavior, dropout rates, and the number of necessary therapy sessions will diminish as a secondary effect of the additional VR element.

Currently, the technology behind the VR element is being developed in parallel with the study, meaning that the development of VR is fluid. This is an asset as it means that the application can be adapted if any technical problems arise. A potential problem that could be encountered and should be resolved as soon as possible is the functioning of the web-based VR application on different smartphones. The application, the videos, and the technology that underlies this must be designed in such a way that it is supported by all types of smartphones and their software versions. To find the right fit for smartphones, testing must be performed on multiple devices before offering them to parents. To minimize the potential obstacles and rather use them as learning points, close contact will be kept with both technical support and parents. Parental feedback will be processed as soon as possible by the technical support, and adaptations will be made where and when necessary. Fortunately, SCEDs lend themselves well to this flexibility, as each individual trajectory has different phases (baseline and treatment phase), thus enabling comparison between phases and even between measurement points. This means that the VR obstacles faced by one family may differ from those of another family. Therefore, this study offers an opportunity to flexibly implement VR and register individual obstacles as they occur, both qualitatively and quantitatively.

The implementation of VR later on in CDI sessions could potentially limit its effect on improving positive parenting skills, as the rate of skill growth from session to session usually gradually declines as families get closer to meeting skill criteria. Nonetheless, the strength of SCEDs designed with phases, randomization, and the opportunity to replicate means that it allows for the comparison of an individual in different phases of treatment. Thus, it allows us to follow progression while also being able to compare the treatment effects of multiple individuals at different times. As analyses are conducted both visually and statistically, a clear overview can be provided for both individual and group treatment effects. This means that both individual and group outcomes can indicate whether the timing of VR implementation affects the effect on acquired positive parenting skills. However, SCEDs also have some potential limitations. For example, there is a risk of type I errors because of the potential violation of distributional assumptions and the presence of serial dependencies. In addition, it is possible that there is an unexpected data trend because of maturation effects (eg, gradual bettering of VR analytics because of habituation to scenarios). With the right statistical support, these pitfalls can potentially be mitigated, which is something the research group intends to do.

### Conclusions

The clinical practice in which the study is conducted lends itself well to the research on VR implementation in this study. This means that every family that participates already provides enough data to be able to say something meaningful about the treatment and VR. Overall, this study can be seen as a stepping stone to implementing VR on a larger scale within PCIT to ultimately magnify the scope of the treatment through additional skill training.

## Abbreviations

AISI: Attachment Insecurity Screening Inventory
CDI: child-directed interaction
CLP: child-led play
CU: clean up
DPICS: Dyadic Parent-Child Interaction Coding System
ECBI: Eyberg Child Behavior Inventory
OBVL: opvoedingsbelasting vragenlijst
OBVL-K: opvoedingsbelasting vragenlijst-kort
PCIT: Parent-Child Interaction Therapy
PDI: parent-directed interaction
PLP: parent-led play
PMT: parent management training
PRIDE: Praise, Reflect, Imitate, Describe, and Enthusiasm
RCT: randomized controlled trial
SCED: single-case experimental design
SPENT: Standard Protocol Items: Recommendations for Interventional Trials extension for N-of-1 trial protocols
TAI: Therapy Attitude Inventory
UMC: University Medical Center
VR: virtual reality
WWC: What Works Clearinghouse
